# Educating for Equity: Preparing Student Midwives for Antenatal Care of Vulnerable Pregnant Women—A Pilot Study

**DOI:** 10.3390/healthcare14070952

**Published:** 2026-04-05

**Authors:** Janice Hill, Tina Werringloer, Ulrike Keim, Maria Meisl, Claudia F. Plappert

**Affiliations:** Department of Midwifery Science, Institute of Health Sciences, University of Tübingen, 72074 Tübingen, Germany; tina.werringloer@med.uni-tuebingen.de (T.W.); ulrike.keim@med.uni-tuebingen.de (U.K.); maria.meisl@student.uni-tuebingen.de (M.M.); claudia.plappert@med.uni-tuebingen.de (C.F.P.)

**Keywords:** antenatal care, circumcision, female, racism, intimate partner violence, trauma, poverty, migration, asylum-seeking, midwifery, students, problem-based learning

## Abstract

**Highlights:**

**What are the main findings?**

- Midwifery students reported higher levels of knowledge and skills after completing an elective course on antenatal care for vulnerable pregnant women.
- The most pronounced changes were reported in the areas of asylum-seeking, female genital mutilation/cutting (FGM/C), and racism.

**What are the implications of the main findings?**

- The findings suggest that focused educational content can support students’ reflection on the care needs of vulnerable pregnant populations.
- Pilot pre–posttest data may inform the further development of midwifery curricula addressing psychosocial and structural vulnerability.

**Abstract:**

**Background:** Maternity care for vulnerable pregnant women presents a particular challenge within midwifery practice. In Germany, maternity services lack standardized frameworks to adequately address the specific needs of individuals who have experienced, among other factors, sexualized violence, poverty, female genital mutilation/cutting (FGM/C), or discrimination. Limited access to healthcare among these populations contributes to increased maternal and neonatal morbidity and mortality. Emerging evidence indicates that comprehensive medical and psychosocial support provided by midwives can substantially improve obstetric outcomes for marginalized pregnant women. **Methods:** An elective course, Antenatal Care for Vulnerable Women, was offered in the sixth semester of the Bachelor’s program in Midwifery Science at the University of Tübingen in 2025. The course provided insights into the psychosocial challenges faced by vulnerable pregnant women and prepared students for these specific aspects of midwifery practice. The curriculum incorporated foundational lectures and innovative teaching formats aimed at cultivating constructivist approaches to problem-solving. All sixth-semester midwifery students were asked to assess their knowledge and skills across five vulnerability categories: asylum-seeking, FGM/C, intimate partner violence, trauma, and racism. A pilot pre–posttest analysis using a 6-point Likert scale (1 = very good, 6 = poor) was conducted as hypothesis-generating and curriculum-guiding. The pretest included 38 respondents. The posttest included 11 respondents who attended the course. **Results:** Students who attended the course demonstrated observable gains in knowledge and skills across all categories, with the greatest improvements in asylum-seeking, median of 5 (IQR 4–5) vs. 2 (2–3); FGM/C, 5 (4–5) vs. 2 (2–3); and racism, 5 (3–5) vs. 2 (2–3). **Conclusions:** Innovative teaching methods may contribute to preparing midwifery students for targeted care of vulnerable pregnant women. Findings from the pre- and posttests provide preliminary insight into the potential value of experiential learning and may inform the further development of practice-oriented teaching methods.

## 1. Introduction

Midwifery care in a complex world requires an understanding of the particular challenges faced by vulnerable pregnant women* (women’s health has historically focused on cisgender women, often excluding transgender and non-binary individuals. In this paper, we include all individuals with reproductive capacity regardless of gender identity). Midwifery science educators are challenged to provide students with the theoretical knowledge and practical skills necessary to prepare them to offer quality care for women confronting psychosocial adversity.

### 1.1. Vulnerability and Pregnancy

Pregnant women in vulnerable situations and their unborn children face increased risks of perinatal mortality and morbidity, including preterm birth, growth restriction, cerebral palsy, thrombosis, cardiac disease, uterine rupture, postpartum depression, PTSD, admission to neonatal and maternal intensive care units [1,2,3,4,5,6].

Vulnerability in pregnancy, however, is not only a matter of health risks, but a complex intersection of personal, societal, and structural factors that shape a woman’s experience of care and her access to support.

To enhance individualized care, midwifery research has sought to categorize and define vulnerability. A European midwifery consortium describes vulnerable pregnant women as those experiencing physical, psychological, cognitive, and/or social risks compounded by limited support and coping capacity [7]. Vulnerability has been indexed through factors such as poor health, language barriers, substance use, limited resources, and family disruption [8]. Broader frameworks conceptualize vulnerability as a complex, lifelong process in which risks are intensified or mitigated by contextual conditions [9]. Rather than viewing vulnerability solely as exposure to adversity (e.g., forced migration, Female Genital Mutilation/Cutting (FGM/C), trauma, legal constraints), it should be understood as a constellation of interconnected events shaped by structural inequalities—including racism, xenophobia, transphobia, and ableism—that restrict access to maternity services. Effective strategies to reach vulnerable pregnant women must therefore address institutional biases that women perceive as barriers to care [9,10,11]. This multidimensional perspective is essential to improving the quality, safety, and equity of midwifery practice. Accordingly, educators must equip student midwives to identify health indicators without stigmatizing marginalized populations, emphasizing that midwifery is not only a clinical practice but also a matter of human rights and cultural competence.

### 1.2. Midwifery Care for Pregnant Women with Complex Psychosocial Needs

Midwifery continuity of care models have shown benefits, such as increased spontaneous vaginal births and reduced cesarean sections, but their effectiveness for women with complex psychosocial circumstances is not fully understood [12]. Successful community-based continuity models of care have been shown to reduce stillbirths, preterm births and the prevalence of small for gestational age infants, as well as increase positive outcomes like skin-to-skin contact, for women with social risk factors [13,14,15]. Targeted care that complements the continuity model for vulnerable women includes culturally responsive care, psychosocial and practical support, assistance navigating health systems, and flexible, accessible services [16]. The World Health Organization advocates for universal adoption of midwifery care models to reduce maternal and newborn mortality and morbidity, emphasizing integration, equity, and human rights-based care, especially for vulnerable populations [17].

Despite the recognized benefits of midwifery care, vulnerable women in Germany face significant organizational barriers to accessing support, highlighting a gap in care continuity and models tailored to their unique circumstances. Currently, there are few designated pathways for the care of women with complex needs, compounded by barriers, such as a lack of awareness about midwifery services and limited availability of midwives for continuous care. Focus group interviews with maternity service users in Germany, including women identified as vulnerable, indicate that women expect midwives to deliver holistic, respectful care and to advocate for their individual needs [18].

### 1.3. Challenges for Midwifery Educators

German bachelor’s degree programs in midwifery are grounded in the International Confederation of Midwives’ (ICM) Essential Competencies for Midwifery Practice, which define the minimum knowledge, skills, and professional behaviors required for entry into midwifery practice. In the 2024 revision, the ICM explicitly strengthened competencies related to equity, human rights, and the provision of respectful, person-centered and rights-based care for women, newborns, and gender-diverse individuals, particularly those living in vulnerable or marginalized circumstances and complex life situations [19]. These competencies emphasize midwives’ professional responsibility to recognize structural vulnerabilities, address health inequities, and provide care that upholds autonomy, dignity, and informed decision-making across diverse social, cultural, and humanitarian contexts. However, these expanded competency domains are not yet explicitly or systematically reflected in the German Regulation on Midwifery Studies and Examinations (Studien- und Prüfungsverordnung für Hebammen, HebStPrV) [20]. This discrepancy highlights the urgent need to integrate the ICM’s rights-based and equity-oriented competencies into national curricula and regulatory frameworks to ensure that midwifery graduates are adequately prepared to meet the needs of diverse and vulnerable populations. Midwifery educators encounter several challenges when developing course content that addresses risk indicators among vulnerable populations, appropriate models of care, and the structural drivers of health inequities. Integrating clinical content with critical perspectives on social determinants of health, systemic discrimination, and implicit bias requires careful calibration, particularly given students’ varied levels of experience. An important consideration is that some students may themselves belong to vulnerable groups or have lived experiences of discrimination or trauma. Consequently, the material may be emotionally charged or personally affecting, necessitating trauma-informed and sensitive teaching approaches.

Educators must also ensure that students learn to identify risk indicators without reproducing stigmatizing assumptions, which demands pedagogical strategies that combine evidence-based practice with critical reflection. At the same time, presenting effective models of care for vulnerable populations often requires drawing on interdisciplinary and context-specific evidence that may be unevenly implemented across health systems. Finally, fostering an environment that supports self-reflection, cultural humility, and open dialogue is essential for engaging with inequities and biases, yet it remains challenging within existing curricular constraints and institutional expectations.

Mindful of these challenges, we designed an elective course entitled “Antenatal care for vulnerable women” for a self-selected group of sixth-semester students in midwifery science at the University of Tübingen. The course was offered from 2020–2025 with an annual cohort of 15–20 students. While course content evolved over time, a core mix of teaching methods was relied upon to generate critical thinking and deeper specific knowledge about the concept of vulnerability and models of midwifery care.

To avoid re-traumatization and triggering, students were given advanced notice of content and provided with an option to opt out.

The elective course comprised 14 sessions. The first four consisted of lectures and discussion, introducing students to social medicine, the social determinants of health, and their relevance to caring for vulnerable pregnant women. Key topics included the effects of poverty, displacement, racism, discrimination, FGM/C and trauma on pregnancy and access to antenatal care. One of these lectures included a presentation from a doctoral student who presented her research about the impact of racism on maternity care. The final lecture discussed successful international models of care and their implementation in the German healthcare system. Elements of the LEGO^®^ SERIOUS PLAY^®^ method [21] are incorporated throughout to facilitate structured reflection.

Subsequently, students went from the lecture hall into the real world in order to speak with women living in complex situations. Two visits in Stuttgart focused on the experiences and needs of asylum-seeking women, some of whom had survived human trafficking and FGM/C, as well as on women who survived trauma from childhood sexualized violence. A third excursion involved an exchange with a local women’s shelter.

The following four linked teaching units used a World Café format [22]. Student representatives prepared guiding questions that small groups discussed before presenting their insights to the full cohort. This structure enabled students to reflect on their experiences and to develop strategies for working with vulnerable pregnant women in their future practice. Because encounters with women affected by violence, poverty, and discrimination may also be emotionally challenging for students, the format supports processing these experiences through dialogue.

The seminar concluded with four consecutive sessions of video-supported, simulation-based role plays [23]. These sessions used video technology and realistic scenarios from midwifery practice—such as antenatal care visits—to allow students to apply their knowledge and skills in a safe, controlled environment. Students assumed the role of midwives, as well as pregnant women. The recorded simulations were then reviewed with an experienced trauma therapist. This process enabled students to assess their performance, reflect on their actions and decision-making, and receive targeted feedback. Debriefings provided space for open discussion, allowing students to explore their reactions, analyze the impact of their decisions, and consider alternative approaches.

As part of their independent study, students created individualized care plans for fictional pregnant women, focusing on a specific vulnerability. All care plans were compiled into a final handbook.

The objective of this study was to assess changes in midwifery students’ capacity to provide individualized, equity-oriented, and rights-based antenatal care for pregnant women living in vulnerable or marginalized circumstances following participation in a targeted educational module.

## 2. Methods

To achieve the study objective, we conducted a pilot pre–posttest study to evaluate changes in midwifery students’ self-assessed knowledge and practical skills following participation in an elective course focusing on antenatal care for pregnant women living in vulnerable or marginalized circumstances. Participants were recruited using purposive convenience sampling, targeting sixth-semester midwifery science students as the population of interest. Participation was voluntary, resulting in a self-selected sample.

Data were collected using a questionnaire adapted from a similar and previously validated instrument developed for medical students [24]. The questionnaire was modified to reflect the midwifery context and the content of the elective course by JH and TW. To enhance content validity, the adapted questionnaire was reviewed by two colleagues from the Department of Midwifery Science who were not involved in the study. The survey showed good internal consistency (α = 0.87) based on 15 items in the present sample.

As the study was designed as a feasibility-based, exploratory pre–post evaluation with a fixed cohort size determined by course enrollment, a formal a priori sample size calculation was not conducted. Ethics approval was granted with these design parameters in place.

In accordance with ethical research standards and applicable data protection regulations, all participant data were anonymized prior to analysis. Personal identifiers were removed, and responses were coded to ensure confidentiality. Data were stored securely and accessed only by the research team. Participants were informed of their right to withdraw from the study at any time without penalty, and written informed consent was obtained prior to participation.

Ethical approval for the study was granted by the Ethics Committee of the University of Tübingen, Medical Faculty (Project No. 228/2024BO2).

Data collection was conducted using a paired pre–post design with standardized online questionnaires on the platform SoSci Survey (Version 3.5.02), comprising five categories to assess theoretical and practical competencies on a 6-point Likert scale (1 = very good, 6 = poor). In the pretest, all students in the 6th semester of the midwifery science program were surveyed in May 2025. Students were asked to self-assess their theoretical and practical knowledge with regard to antenatal care of vulnerable pregnant women in the categories: intimate partner violence, FGM/C, asylum-seeking, trauma and the experience of racism. A teaching intervention took place between the pre- and posttest. The posttest was administered only to participants of the module in July 2025. Response rates were 84% (*n* = 38/total *n* = 43) for the pretest and 65% (*n* = 11/total *n* = 17) for the posttest. Matching of pre- and post-test responses was achieved through the use of pseudonymous participant codes. Rating of the items was based on a 6-point Likert scale, analogous to German school grades (1 = very good, 6 = poor). The Likert scale provides ordinal data with a natural ranking; nonparametric measures (median and interquartile ranges (IQR)) are reported. Because pre- and posttest responses could be linked at the individual level, changes in outcomes were analyzed using the Wilcoxon signed-rank test. The results were analyzed with IBM SPSS Version 31.

## 3. Results

Students identified their gender as 97% (*n* = 37) female and 3% (*n* = 1) diverse. All participants (*n* = 38) had completed the German general university entrance qualification (Abitur). In addition, 16% (*n* = 6) had completed a prior Bachelor of Science and 3% (*n* = 1) the German dual vocational training/apprenticeship (Ausbildung).

**Figure 1:**
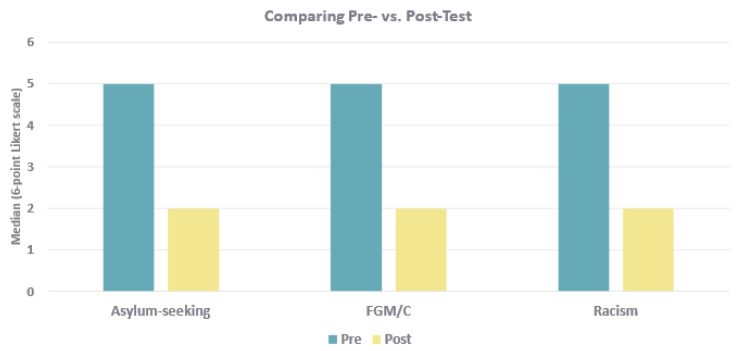
Pre and Posttest Self- Assessment of Knowledge and Skills in Antenatal Care of Vulnerable Women. Pre-test (*n* = 38) completed by all sixth-semester students; posttest (*n* = 11) completed by students in the same cohort who attended the module “Antenatal Care of Vulnerable Women.” Rating based on a 6-point Likert Scale (1 = very good; 6 = poor). Lower scores indicate higher perceived knowledge and skills. All categories showed improvement, with the greatest gains in asylum-seeking, Median 5 (IQR 4–5) vs. 2 (2–3); FGM, 5 (4–5) vs. 2 (2–3); and racism, 5 (3–5) vs. 2 (2–3).

All categories showed marked improvements in median values between the pre- and posttest. The most pronounced changes were observed for care of asylum-seeking women (median: 5 [interquartile range 4–5] vs. 2 [2–3]), women with FGM/C (5 [4–5] vs. 2 [2–3]), and recognition of the effect of racism on perinatal health (5 [3–5] vs. 2 [2–3]). Less pronounced changes were found for Intimate Partner Violence (IPV) (4 [3–5] vs. 3 [2–3]), experiences of trauma (3 [3–4] vs. 2 [1–3]), and general understandings of vulnerability and its impact on maternal health (3 [2–3] vs. 2 [1–3]). These results indicate an observable increase in knowledge and/or a change in attitudes as a result of the intervention, as shown in Figure 1.

## 4. Discussion

To provide high-quality antenatal care for vulnerable populations, midwives must be able to identify health risk factors, develop strategies to address barriers to access, demonstrate cultural competence, and understand how structural and institutional biases intersect with individual adverse circumstances. Given ongoing personnel shortages and limited organizational capacity to develop models for complex care [11,18], our findings underscore the importance of equipping midwifery students with these competencies during pre-service education.

This study builds on a five-year implementation of the elective course “Antenatal Care for Vulnerable Women” in the sixth semester of the midwifery program at the University of Tübingen. The pre–posttest findings provide insight into how targeted educational interventions may contribute to competence development in this area.

The most pronounced improvements were observed in the areas of asylum-seeking, FGM/C, and racism, which coincided with students’ direct dialogue with affected women through partner organizations. This finding suggests that experiential and dialogical learning constitutes a key mechanism underlying competence development in these domains, a conclusion supported by previous research highlighting the value of engagement with lived experience in midwifery education [25,26]. Less pronounced changes in areas such as intimate partner violence and trauma may reflect knowledge already acquired in earlier modules, pointing to the benefit of integrating content on vulnerability longitudinally across the curriculum rather than confining it to elective formats.

Across all teaching formats—LEGO^®^ SERIOUS PLAY^®^, World Café discussions, and simulation-based role play—structured reflection and experiential learning enabled students to process emotionally challenging content, enhance perspective-taking, and translate theoretical knowledge into practice. Rather than functioning as isolated pedagogical tools, these formats collectively supported students’ emotional awareness, sense of agency, and capacity for reflexive, equity-oriented care.

To mitigate students’ feelings of helplessness when confronted with the lived experiences of vulnerable women, it was important to cultivate their sense of agency in shaping care. The World Café format provided a structured opportunity for students to critically engage with course content and collaborate on building innovative models of care that are applicable across the entire childbearing continuum [22].

Collaborative role-playing allowed students to use their theoretical knowledge to construct situations that would test their communication skills, cultural competence and clinical ability. By creating their own scripts and scenarios, students were able to slip into the roles of both the client and the midwife, enhance their sense of empathy and test the efficacy of care plans [23,27].

Not all aspects of vulnerability could be covered in the module, e.g., maternity care for the queer community. We addressed this gap by requiring students to develop an individual care plan for a fictitious pregnant person, which allowed students a self-directed learning experience.

In a routine university evaluation of the module, with both open and closed responses (EvaSys, University of Tübingen, Medical Faculty), students reported substantial educational benefits, including perceived gains in existing and newly acquired competencies. Participants further emphasized that, given the frequency with which vulnerable women are encountered in clinical practice, training in this area should be integrated more systematically into the curriculum rather than remaining an elective.

The relatively small sample size limits the extent to which these findings can be generalized to the broader population of students in midwifery science. Moreover, the subset of students who completed the posttest was subject to a high risk of selection bias, as participation in the course was voluntary and therefore self-selected. Reliance on self-reported measures may have also introduced social desirability bias, potentially influencing how participants reported their experiences. The sample also showed a highly homogeneous gender distribution, which precluded meaningful analysis of gender-related differences; gender identity was therefore reported descriptively as contextual information relevant to the study focus rather than as an analytic variable. Additionally, interpretation of the pre–post findings should also account for posttest attrition, as some students could not be included in the longitudinal analysis due to inconsistently entered self-generated pseudonymous identifiers at follow-up, which precluded reliable data matching. This may have reduced statistical power and introduced selection effects, underscoring the need for more robust yet anonymity-preserving identification procedures in future evaluations. Finally, it was not possible to assess potential confounding variables—such as students’ trauma histories or experiences of discrimination—due to ethical and privacy considerations. Internal validity was good, despite the small sample size.

Taken together, the findings should be interpreted cautiously. While the observed changes are consistent with the intended learning objectives of the module and align with existing literature on experiential learning, the study design does not permit causal inferences regarding the effectiveness of specific pedagogical components. Self-selection into the course, reliance on self-reported outcomes, and the inability to account for prior experiences or positionality likely shaped both baseline scores and observed changes. Nevertheless, the convergence of quantitative trends, qualitative feedback, and theoretical alignment suggests that targeted educational interventions addressing vulnerability hold promise, warranting further investigation using larger samples and more robust study designs.

## 5. Conclusions

This pilot pre–posttest study examined changes in self-reported competencies among midwifery students participating in an elective module focused on antenatal care for women living in vulnerable circumstances. The findings indicate changes across several competence domains following course participation, particularly in areas addressed through dialogical and experiential learning components. Students also expressed strong engagement with the topic and interest in developing context-sensitive approaches to care for pregnant clients facing complex psychosocial challenges.

While the study design does not allow causal attribution or conclusions regarding clinical performance, the observed patterns suggest that participation in the module was associated with increased awareness of social, cultural, and structural dimensions of vulnerability. Students’ responses point toward an emerging understanding of vulnerability as shaped by broader social and institutional contexts rather than solely by individual characteristics. These interpretive trends should be understood as exploratory and require further investigation using larger samples and more robust study designs.

Given its pilot nature, this study primarily serves to inform the design of future research and curriculum development efforts. The pre–posttest approach enabled the assessment of within-cohort change and highlighted areas where targeted educational content may be perceived as particularly relevant by students. Future studies should build on these findings by incorporating comparison groups, longitudinal follow-up, and additional outcome measures to more precisely assess the impact of specific pedagogical strategies on competence development in midwifery education.

## Data Availability

The data presented in this study are not publicly available due to ethical restrictions and data protection regulations. The dataset contains information derived from human participants collected under ethics approval that does not permit public sharing of raw data. Aggregated data supporting the findings of this study are available from the corresponding author upon reasonable request.
